# Supplementary figures and images for: Biodegradable Scaffolds for Vascular Regeneration Based on Electrospun Poly(L-Lactide-co-Glycolide)/Poly(Isosorbide Sebacate) Fibers
Source: Int J Mol Sci. 2023 Jan 7;24(2):1190. doi: 10.3390/ijms24021190 (PMC9866311; doi:10.3390/ijms24021190)

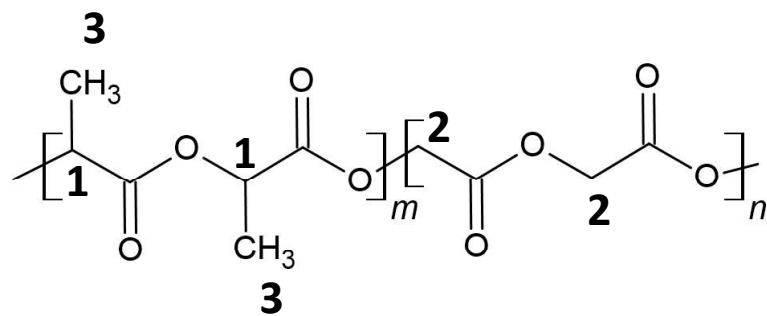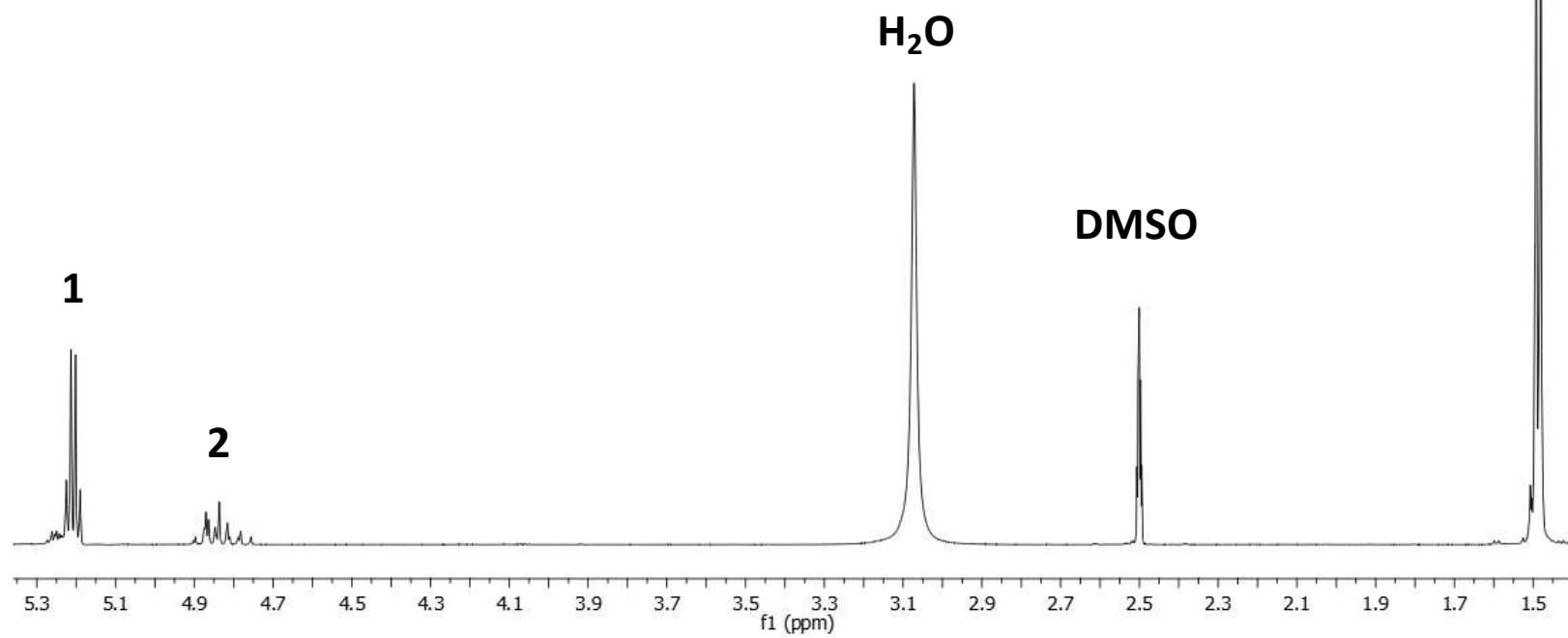

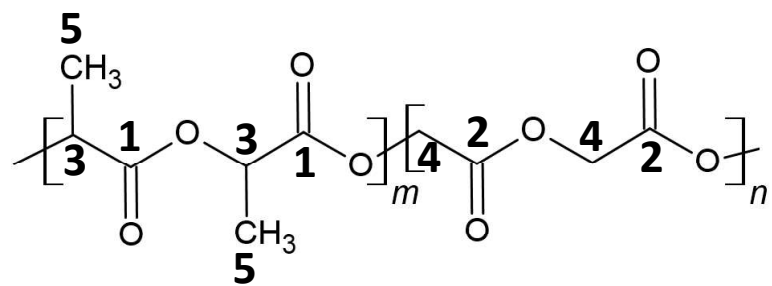

DMSO

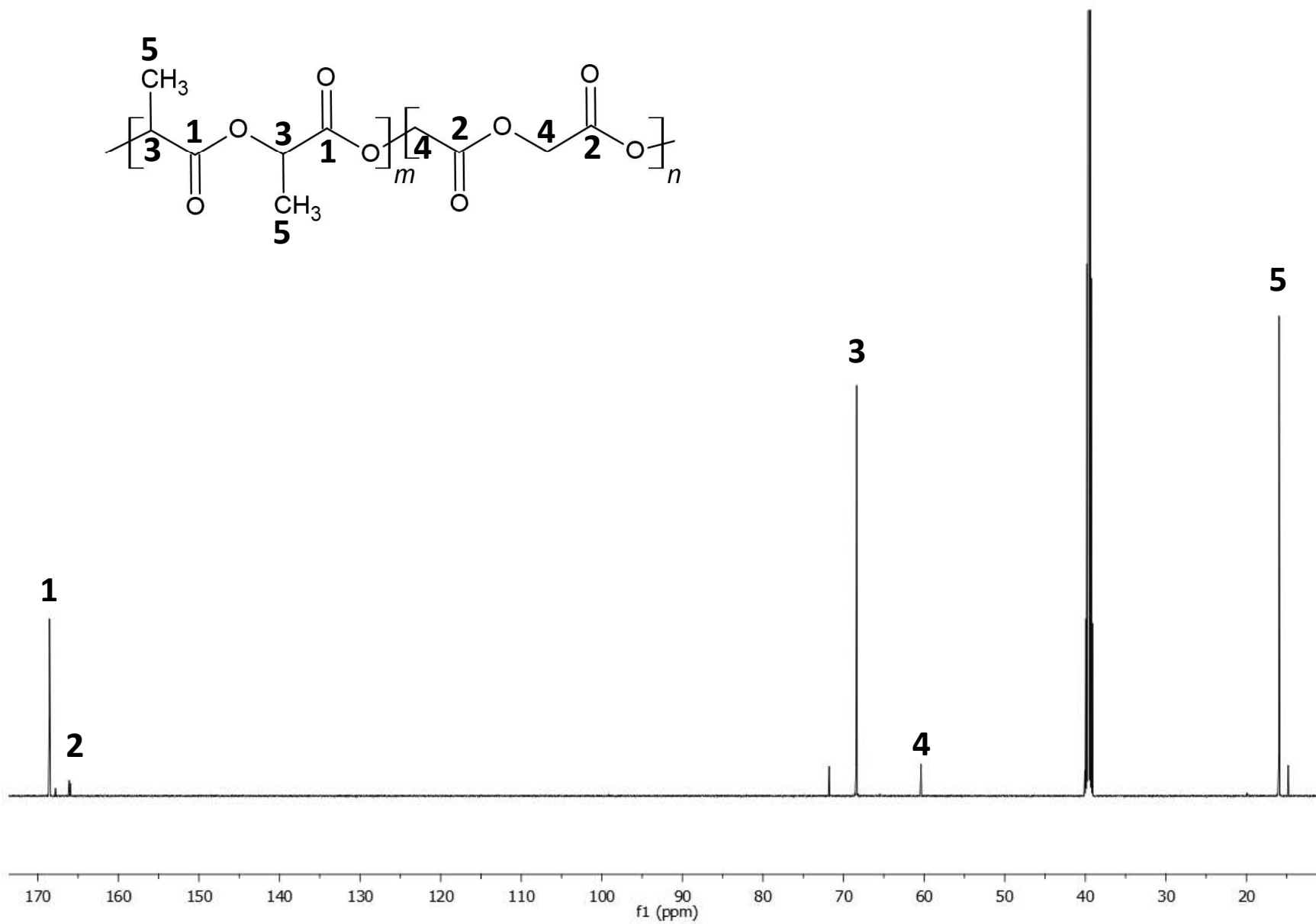

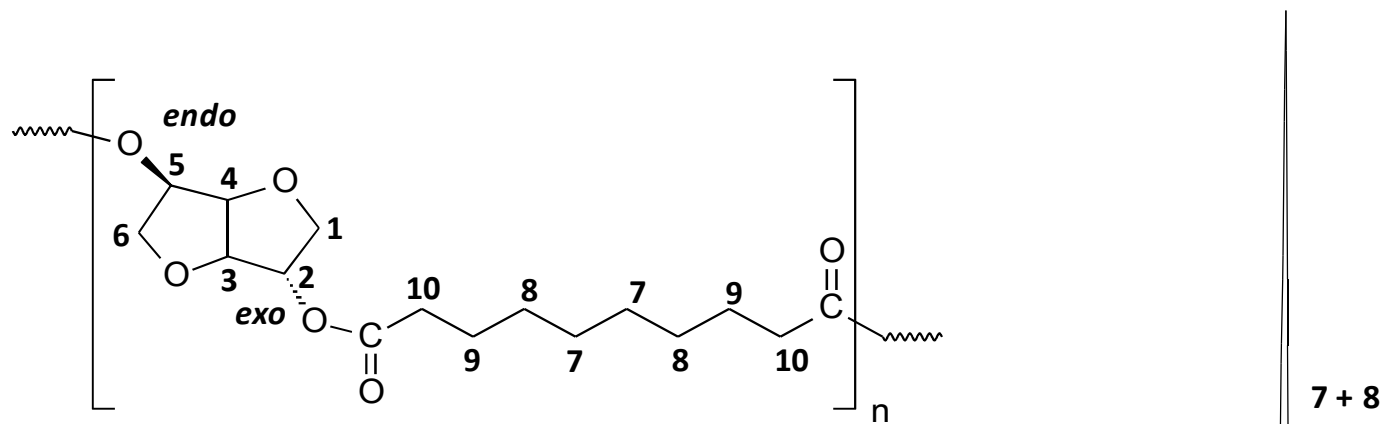

10 *endo*, 10 *exo*

1a,1b

6b

1a

2 + 5

4

3

9

7 + 8

5.8 5.6 5.4 5.2 5.0 4.8 4.6 4.4 4.2 4.0 3.8 3.6 3.4 3.2 3.0 2.8 2.6 2.4 2.2 2.0 1.8 1.6 1.4 1.2 1.0 0.8 0.6

f1 (ppm)

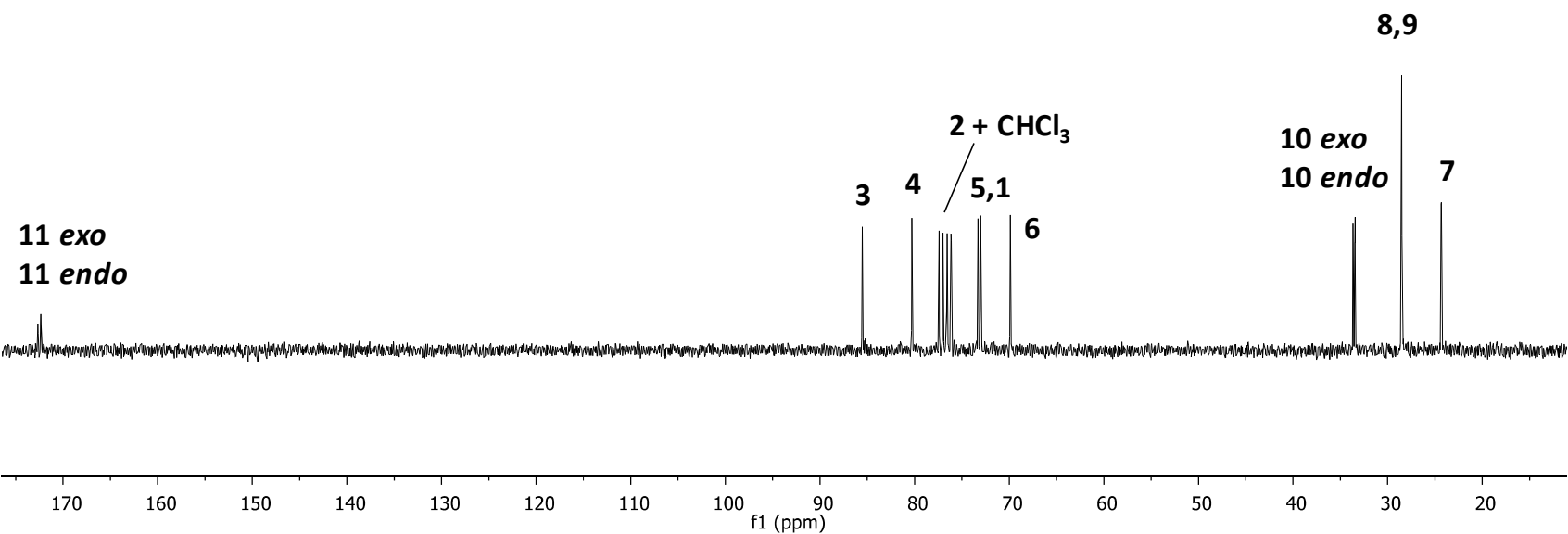

Supplement: Supplementary file 1 [file ijms-24-01190-s001.zip › ijms-2100104-supplementary.pdf]
